# The Adapting Mind in the Genomic Era

**DOI:** 10.3389/fpsyg.2016.00078

**Published:** 2016-02-02

**Authors:** Martin Fieder, Susanne Huber

**Affiliations:** Department of Anthropology, University of ViennaVienna, Austria

**Keywords:** behavioral anthropology, evolutionary psychology, human genetics and genomics, bioinformatics, epigenetics

## Abstract

Genomics and molecular biology has added substantial methods and knowledge to nearly all fields of biology and medicine. In this review we try to demonstrate how genomics and molecular biology is also on the way to have a profound impact on behavioral anthropology, evolutionary psychology, evolutionary sociology, and bio-sociology. We propose that particularly studies on “selection and adaptation” will be influenced profoundly by genomics, for instance via identification of the partially genetic basis of human behavior by “candidate gene studies” and by “genome wide association studies.” In addition, epigenetics will lead to a deeper study of the interaction of the genetic basis of a behavior and its environmental regulation. We argue that the “genomic revolution” is much more than merely a new methodological approach, but will change our concepts of human behavior and its development in the evolution of homo.

## Introduction

Evolutionary studies on human behavior—not always free of friction—contributed substantially to the “unification knowledge” in the way of “consilience,” proposed by Wilson (1999), one of the founders of socio-biology and one of the most important contributors to the field.

Evolutionary studies on human behavior helped to shed light on so important questions as the emergence of human cooperation and morality, economics, human aggression, the explanation of human mating patterns, human reproduction and demography, the importance of social status, the evolution of culture, and many more.

Concurrently with evolutionary studies on human behavior that revolutionized ideas and concepts of human behavior, “*the genetic and genomic revolution*” took place. It has received less attention by evolutionary psychology and behavioral anthropology. Nevertheless, this field is expected to have dramatic consequences for all studies on human behavior and human psychology.

Twenty years ago, at the time “The Adapted Mind” (Barkow et al., 1995) was published, genomics was a nascent field. Sequencing of nuclei-acids was very time consuming and very costly. Moreover bioinformatics, a key field in this context, was in a stage of early development. Now, 20 years later, the situation has changed fundamentally: Since the “start” of the human genome project ~25 years ago (Olson, 1993), the sequencing of human genomes has improved manifold both in accuracy and speed: the time necessary to sequence a single human genome shrank from years to months to a couple of days. The results of these efforts cumulated in many significant databases of completely sequenced human genomes, the “1000 genomes” database probably being the most prominent and important among them. Albeit the 1000 genomes database does not offer any phenotypical information (other than sex, ethnicity, and some intra-family relationships), the database (http://www.1000genomes.org) does provide access to the completely sequenced genomes of over 2500 individuals from 25 populations worldwide, thereby enabling also studies on human genomic adaptation. This database is extraordinarily suitable to detect signs of selection in the human genome. It also offers the possibility to download the complete genomic information of currently 2504 individuals from 25 populations from around the world. The definition of standard formats such as the variant call format (VCF http://www.1000genomes.org/data#DataAvailabl) enables an easy and fast access to genomic data from individuals, populations, and from continents via the download of either whole genomes, the sequence of chromosomes, or individual genes. Together with raw sequence information this facilitates even the precise finding of single nucleotide polymorphisms (SNPs, which are genomic differences on a single nucleotide basis, i.e., A, T, C, or G). Table 1 lists the most important data sets and their potential uses.

**Table 1:** **Selection of genomic data bases**.

**Data Base**	**URL**	**Genomic Information**	**Potential Application**	**Aavailability**
1000genomes	http://www.1000genomes.org/	The depth sequenced genome of 2504 individuals from 26 populations worldwide including also about 800 couples and their offspring	Essential basis for nearly every research question in human genetics. Importance for Evo. Anthro: selection detection, admixture detection, studies on mating and genome	Completely free
UCSC Genome Browser	http://genome-euro.ucsc.edu	Reference sequence of many species as well as of the Neanderthal Project	Basis for genome studies particularly across the borders of species	Completely free
Personal Genome Project	http://www.personalgenomes.org/Harvard	Genomes of volunteers (mainly SNPs), information on phenotypes (mainly medical data such as medical records, measurements, MRI images etc.)	Genetic variation, candidate gene approaches, genome wide association studies	Completely free
Health and Retirement Study	http://hrsonline.isr.umich.edu/index.php?p=gwas&jumpfrom=DD	Genomic data (3 million SNPs) of 20,000 respondents plus surveyed phenotypical information	Genetic variation, candidate approaches and genome wide association studies particularly on social and behavioral variables, and studies on mating and genome	Proposal and approval of ethic commission necessary
Wisconsin Longitudinal Study	http://www.ssc.wisc.edu/wlsresearch/documentation/	More than 100 SNPs plus longitudinally surveyed phenotypical data	Candidate gene approaches particularly on social and behavioral variables	Proposal and approval of ethic commission necessary
Avon Longitudinal Study of Parents and Children	http://www.bristol.ac.uk/alspac/participants-old/genome/	DNA data of 4000 individuals, plus surveyed phenotypical data	Genome wide association studies and candidate gene approaches	Applying a research proposal
National Longitudinal Study of Adolescent to Adult Health (Add Health)	http://www.cpc.unc.edu/projects/addhealth	Genomic data of DAT1 (dopamine transporter), DRD4 (dopamine receptor), SLC6A4 (serotonin transporter), MAOA_V (monoamine oxidase A-uVNTR), DRD2 (dopamine D2 receptor), and CYP2A6 (cytochrome P450 2A6), DRD4 (dopamine receptor), MAOA (monoamine oxidase A-uVNTR) and 5HTTLPR (serotonin transporter), HTTLPR La-Lg-S, triallelic	Candidate gene approaches	Ethic commission approved security plan for handling and storing sensitive data

The tremendous amount of free available genomic data and a variety of tools make it possible for the first time to quantitatively measure selection directly in the human genome (for details, see below) instead of phenotypically describe and measure selection and adaptation. We posit that this is of particular interest for “measuring selection” in genes associated with human behavior. Moreover, the process of ever-improving sequencing is ongoing: new devices of smart-phone size are under development that will enable researchers to sequence human genomes within hours at very low costs and under “field conditions” (https://www.nanoporetech.com).

Also data bases offering genetic and phenotypic information are rapidly developing. For instance the health and retirement study (HRS–http://hrsonline.isr.umich.edu/index.php?p=data) is currently one of the most comprehensive databases linking genetics and phenotypical information. That study surveyed various aspects of the life course of over 20,000 individuals from the US and has to date genotyped 20,000 individuals (about 1.5 million SNPs). This makes it a prime candidate for research on “Genome Wide Association Studies” (GWAs) for the identification of the genetic basis of behavioral traits.

Without any further restriction, the personal genome project (http://www.personalgenomes.org/harvard) provides genomic and trait data from volunteers. Although, particularly the phenotypical information is less systematic in this database, the personal genome project offers a very rich and fascinating collection of highly valuable information, including for some individuals also the microbiome. Before the data can be used, however, they require some processing work.

Overall, the field of genomics has developed very rapidly from a field with a very high laboratory effort to one providing “tons of free data” also for use by scientists who may not be able to cover the expenses of whole genome sequencing.

Overall, in our opinion genomics has the potential to substantially change the field of behavioral anthropology and evolutionary sociology and biosociology.

The potential impact of genomics has also been outlined by Bolhuis et al. (2011), who criticizes several views and discusses new opportunities for evolutionary psychology, amongst others the prevalence of recent selective sweeps in human evolution, that counters the arguments of representatives of evolutionary psychology.

### Selection and adaptation

Tooby and Cosmides (1989) presumed that “the complex architecture of the human psyche can be expected to have assumed approximately modern form during the Pleistocene, in the process of adapting to Pleistocene conditions and only undergone minor modifications since then” and that “there is no a priori reason to suppose that any specific modern cultural or behavioral practice is adaptive” (Symons, 1990), suggesting, that evolution has nearly stopped after the Pleistocene, and therefore “measuring reproductive differentials is at best an inefficient and ambiguous way to illuminate adaptation” (Symons, 1990). These arguments are clearly in opposition to the view of behavioral ecology. Turke (1990a), for instance, argues that “studies of current reproductive consequences (adaptiveness) can help us understand the extent to which psychological mechanisms are general purpose.” Also Irons (1990) and Betzig (1989) argued in the same direction, contradicting Symons' opinion that the environment of evolutionary adaptation should be limited to Pleistocene societies (Symons, 1990).

We are clearly in favor of the arguments of “behavioral ecology,” agreeing that measuring fitness effects beyond the Pleistocene is of crucial importance for the understanding of adaptations. We propose that genomics, which offers a sophisticated toolset to directly test for selection, may provide a new additional means of measuring both past and possibly still ongoing adaptations. Together with studies on reproductive consequences of a trait, this approach will enable to make claims about adaptations in humans on empirically well-founded grounds.

Accordingly, in our opinion, the view that there would be hardly any ongoing selection in humans, as co-adapted gene complexes would be unable to respond quickly to selection (Tooby and Cosmides, 1989), seems to be outdated. We see no reason to assume that the process of adaption should have stopped in the Pleistocene so that most of our adaptations date back “a long time,” and we thus should not have been able to adapt to rather recent environments. In fact, there are various examples showing that adaptation has been taking place after the Pleistocene, the most prominent ones being the case of the lactose tolerance (Beja-Pereira et al., 2003; Burger et al., 2007), or the case of the copy number variations in the salivary amylase gen which indicates differences between populations on high-starch compared to low starch diets (Perry et al., 2007), both of which are also good examples for culture-gene co-evolution. In line with this, Cochran and Harpending (2009) made the point that the development of agriculture has in fact “increased the rate” of human evolution.

Albeit effect sizes are small, there is also evidence that natural selection is acting on body height even in contemporary human populations, which however does not mean that selection did not act in the past (Stulp and Barrett, 2016). Interestingly, also ongoing germ line selection processes may take place: Kong et al. (2004) reported that in mothers with a high oocyte recombination rate, there is a higher chance that a gamete becomes a live birth, so that mothers having a higher recombination, tend to have more children. Accordingly, the authors concluded, that not only genomic variants but also recombination itself might be under selection. Methods developed by modern statistical genetics now enable us to detect such signs of ongoing selective processes (please find a detailed description below). For instance, signs of recent selective sweeps in the whole genome had been extensively documented on the basis of the hapmap data by van Voight et al. (2006). In addition, Williamson et al. (2007) showed that about 10% of the human genome is affected by a linkage to relatively recent selective sweeps, the phenotypical association of genes (respectively loci within genes) ranging from forming protein complexes, olfactory receptors, immune system genes, heat shock genes, to genes involved in the development of the nervous system. If so many genes have undergone selective sweeps, we cannot see any serious reasons why also genes associated with behavioral traits should not have been recently or even currently under selection.

Additionally, genetic differences among individuals which are associated with behavioral traits should not be underestimated. Of course, it depends upon definition, what should be viewed as genetic difference among individuals. Differences may be important whenever they exert any influence on a trait, i.e., whenever a trait varies due to genetic differences between individuals. In this respect, even the difference at one single locus can result in profound phenotypic differences. Well-documented examples of an association between genomic variation on a single nucleotide basis and behavioral traits are MAOA (monoamine oxidase A) and aggressive behavior (Kim-Cohen et al., 2006), COMT (catechol-O-methyltransferase) and the modulation of stress sensitivity (Zubieta et al., 2003), OXTR (Oxytocin receptor gene), and social behavior (Ebstein et al., 2012). Other examples are SNPs associated with height (Yang et al., 2010), which may help to investigate evolutionary questions on human height (Stulp and Barrett, 2016) on the basis of genomic data.

Certainly, as discussed above, the adaptive value of a phenotypical trait can also be investigated by analyzing the association between that trait and its fitness consequences (Betzig, 1989; Irons, 1990; Turke, 1990b). In the case the association between a gene/genetic locus and a particular trait is “straight forward” and rather simple, we therefore, assume that a “dual strategy” might be fruitful, investigating both whether this trait has reproductive benefits, and whether traces of selective sweeps can be found at this gene/locus. Currently such a “dual approach” will however, only be possible for rather simple gene-phenotype associations. Whereas, for the usually much more complex scenarios of multigenic and/or pleiotropic (one gene influences more than one trait) associations, it will be hard (if not impossible) to determine the reproductive advantages and traces of selective sweeps associated with these genes/loci, with the help of the tools recently available We therefore, propose for the moment to focus on rather simple genome–phenotypic associations to investigate whether selection has been acting (or is acting) upon a particular trait and whether it is associated with recent reproductive benefits.

The Oxytocin receptor gene may be an interesting gene for such a study design. Among other functions, the neuropeptide oxytocin plays an important role in modulating cognition and social behaviors (Ebstein et al., 2012; Westberg and Walum, 2015). The neurophysiological effects of oxytocin are shown in facilitating social communication, affiliative behaviors, and social cognition. Oxytocin has been reported to influence maternal behavior, trust, generosity, empathy, eye contact, face memory, and also to reduce anxiety (Insel, 2010). The corresponding receptor gene (OXTR) is expressed mainly in the hypothalamus. Recently, polymorphisms in the intron of the OXTR gene were reported to be associated with various aspects of social cognition and social behavior. The SNP rs53576 on OXTR, for instance, apparently influences human empathy (Rodrigues et al., 2009). Another SNP, rs7632287 on OXTR plays a role in pair-bonding (Walum et al., 2012). SNPs on OXTR also seem to play a role in pro-social behavior. In experiments, Israel et al. (2009) demonstrated that the SNPs rs1042778, rs2268490, and rs237887 on OXTR intron 3 are associated with performance in the dictator game and the social value task. As Schaschl et al. (2015) recently found signs of strong directional selection on another SNP on OXTR (rs59190448, associated with early life stress and anxiety, Myers et al., 2014), which is in high Linkage Disequilibrium (*LD, indicating the non-random association of alleles at different positions; although this is not statistically correct, it could be best understood as if alleles were “correlated”*—*a detailed explanation of the concept of LD, would go far beyond this text, a very good explanation can be found in* Foulkes (2009) with further SNPs on OXTR with various known phenotypical associations (e.g., pro-social behavior, mental diseases etc.), it could be speculated, that the selective force on OXTR may be induced by the increasing necessity of social behavior during human evolution.

This view is supported by the fact that at least in men pro-social behavior leads to increased resource availability and higher social status, (Gurven, 2004) as well as reproductive benefits (Fieder and Huber, 2012). We thus, speculate that the fitness increase for those who behave more pro-socially may have eventually led to an increase in the allele frequency of those alleles associated with pro- social behavior, as for instance those on the OXTR gene. Nevertheless, at present this conclusion remains very speculative, as we neither know the exact physiological functioning of the SNP under selection on OXTR, nor do we know how multigenic/pleiotropic pro-social behavior is (from twin studies is only known that pro-social behavior has a strong genetic basis; Ebstein et al., 2010). Studies are needed to clarify whether rs59190448 is related to any other behavior or trait apart from its association with early life stress and anxiety.

Another example is the case of social status. On the basis of data from traditional societies as well as modern societies, many studies find a positive association between male social status and offspring number (Borgerhoff-Mulder, 1988; Chagnon, 1988; Fieder et al., 2005; Hopcroft, 2006, 2015; Fieder and Huber, 2007; Nettle and Pollet, 2008; Barthold et al., 2012). If the investigation of the genomic background of social status would lead to the identification of associated loci, it would then be possible to search for signs of ongoing selection on these loci (using particular tools able to detect signs of ongoing selection such as or instance the extended haplotype estimation). However, one should not expect a “direct association” between genes/genetic loci and social status, but a link between personality characteristics favoring social status. Accordingly, Vall et al. (in press) found that all personality dimensions are under sexual selection but moderately different between the sexes. Sexual selection largely acts in males through the acquisition of wealth and through the time of engagement, possibly giving reproductive advantage to males high in persistence-compulsivity (Vall et al., in press). Hence according to Vall et al. (in press) it would, for instance, be reasonable to search for signs of selection on these and other loci related to personality, rather than looking for “direct selection” on social status.”

Overall we propose a “dual search” for the adaptive value of a trait by (i) the investigation of reproductive benefits associated with the trait as well as (ii) the “proof “of selection in the genome. Together both may lead to more “mature” conclusions about selection on social status (Nettle and Pollet, 2008).

## Methods of selection detection

Various selection detection approaches exist. At present, the most comprehensive data base for the detection of selection provides 1000genomes project (meanwhile 2504 genomes from, 26 populations-http://www.1000genomes.org/). To reduce the risk of false positive results we strongly recommend the use of several different selection detection algorithms in order to demonstrate that a gene/locus is under selection.

Overall, based on genomic data, two central types of selection can be detected with the methods we are proposing in the following section:

- Directional selection: A phenotype is favored over other phenotypes, leading to a shift in the allele frequency for those alleles associated with this phenotype. Hence the advantageous allele/alleles increase in frequency due to the differences in survival and reproduction of the favored phenotype
- Balancing selection: a selective process in which no phenotype is clearly favored; multiple alleles remain active in the gene pool. This is most common if the heterozygous allele/alleles have a higher adaptive value compared to the homozygote allele/alleles.

The information provided by selection detection algorithms is usually much more abstract compared to that provided by “classical behavioral studies.” Such algorithms “prove,” for instance, in the case of directional selection that an increase in frequency of allele A over allele B from population Y vs. population X (if we assume that X is ancestral) is caused by a selective event. In some cases a phenotypic association is known for a certain allele (mostly SNPs—single nucleotide polymorphisms, e.g., the variation of single base- pairs in DNA sequence).

A possible workflow of using different approaches of selection detection is outlined in Schaschl et al. (2015). Typical outlier approaches are LOSITAN (http://popgen.net/soft/lositan/; Beaumont and Nichols, 1996) and Bayescan (http://cmpg.unibe.ch/software/bayescan/; Foll and Gaggiotti, 2008), a Bayesian approach, which are able to detect signs of selection no older than 80,000 years (Oleksyk et al., 2010). Both approaches test selection based on extremely high *F*_ST_-values (a measure of population differentiation) for positive directional selection, and extremely low *F*_ST_-values for balancing selection. LOSITAN is based on the approach that genetic loci may be assumed of being under selection whenever they show either a particularly high or particularly low differentiation, quantified by common *F*_ST_ statistics: if the *F*_ST_-value at a certain locus is higher (directional selection) or lower (balancing selection) than the expected heterozygosity, this locus is assumed (indicated by a *p*-value) of being under selection. LOSITAN plots the results by separating loci under directional selection, neutral loci (loci under no selection) and loci under balancing selection via confidence intervals.

Like LOSITAN, Bayescan also uses *F*_ST_ calculations, however on the basis of a Bayesian statistical approach: Bayescan calculates an “alpha value,” indicating diversifying selection if it is positive and balancing selection if it is negative.

In addition to outlier approaches, completely different methodological tools could also be used to achieve even stronger confirmation of the results, as for instance the “extended haplotype structure” to detect directional selection (detailed description in (Sabeti et al., 2002); and (van Voight et al., 2006), but implemented this year in SelScan https://github.com/szpiech/selscan–Szpiech and Hernandez, 2014).

The concept of Selscan is based on the “*extended haplotype structure*” and the model of “*hard selective sweeps*” (i.e., mutations arising “*de novo*” on a haplotype are in high LD with the genomic area in the “neighborhood”). Before Selscan can be applied, the haplotypes of a certain genetic region have to be estimated statistically, typically using programs such a SHAPEIT (https://mathgen.stats.ox.ac.uk/genetics_software/shapeit/shapeit.html), which estimates haplotypes on the of basis genotype data (i.e., diploid genomic data) known as phasing. A signal of high haplotype homozygosity arises if selection is strong enough (i.e., the mutation provides selective benefits), and selection occurs faster than recombination acts to break up the LD structure of a haplotype. So an allele with an unusually long-range LD structure compared to its population frequency provides a signature of positive natural selection (Sabeti et al., 2002). In Selscan, three of the most common measurements of the “extended haplotype” are implemented: the Extended Haplotype Homozygosity (Sabeti et al., 2002), the Integrated Haplotype Score (iHS) and Cross-population Extended Haplotype Homozygosity. For a detailed description of each measurement please see Sabeti et al. (2002), van Voight et al. (2006), and Sabeti et al. (2007). The “extended haplotype structure approach” can also detect recent or on-going selective events (not older than ~30,000 years–Oleksyk et al., 2010) using a single population (van Voight et al., 2006).

Concerning the interpretation of the results, if you are “lucky,” you may find a selection pressure on an allele with known phenotypical associations by candidate gene studies or genome wide association studies (explained below), which would simplify the interpretation of your finding. If you detect a selection pressure on a locus without known phenotypical association, then the next step is to search for loci in high LD with a known phenotypical association.

### Candidate gene approaches

Candidate gene approaches to behavior have been the most common methodology in behavioral genetics. They involve sampling of both behavioral and genetic data as well as statistical analysis. Most important for candidate gene approaches is a prior knowledge about which genes are potentially involved in the respective behavior. Also, important is knowledge of the physiological functioning on the gene level as well as of the neurological and hormonal basis of the behavior. A better understanding of the physiology of gene functioning would be a great progress, though currently, our knowledge is mostly only sparse and remains one of the most important research questions in humans genetics (reviewed for instance in Pierce, 2013). Certainly, candidate gene approaches are particularly complicated in traits influenced by many genes or genetic regions (multi genic traits) or if a gene influences more than one trait (pleiotropy), also causing a limitation for GWA studies (see below). This seems to hold true particularly for complex traits such as for instance educational attainment (Rietveld et al., 2013) or the genes influencing the form of the human face (Liu et al., 2012).

Anyway, a good starting point for candidate gene approaches is to include loci previously found to be under selection as it has been demonstrated that loci under selection may be particularly involved in morphological and disease related phenotypes contributing to the diversity of human populations (Barreiro et al., 2008). We assume that this may also hold true for “behavioral phenotypes.”

Also the approach of searching for signs of selection in a gene/loci that had been identified of being functionally associated with a certain trait could be another fruitful approach because the function of this gene/loci might have developed according to the selective forces operating in the past. So in the case that such a selective sweep does not date back too long in time, we may find signs of selection.

In studies on behavioral genetics, for example, experiments (such as the “dictator game” —e.g., one player, “the dictator,” determines how to split an endowment between her/him and another player) are performed, and the genetic region suspected of being related to a behavioral trait is genotyped for each of the experimental subjects. Statistically the data are analyzed by regressing (usually by a GLM) the genotypes of (of certain alleles) on the quantified outcome of the behavioral experiments. This quantifies the association between alleles and behavioral outcomes. This statistical procedure is usually followed by an extensive “interpretation-step” to determine the potential genetic and physiological functioning of the found alleles using information available from various bioinformatics sites (particularly genome browsers: http://genome-euro.ucsc.edu, http://www.ensembl.org/index.html). Further laboratory investigations can also be conducted on the functioning of the found alleles.

An illustrative example of a successful candidate gene approach can be found in Israel et al. (2009), studying polymorphism on OXTR and behavior in the dictator game. Further examples are the discovery of the association of polymorphisms on the dopamine transporter (DAT1/SLC6A3), the serotonin transporter (5HTT), and pathological criminal behavior on basis of data from the National Study of Adolescent Health, that additionally suggest a gene environment interaction (Vaughn et al., 2009). Also on the basis of the National Study of Adolescent Health, Guo et al. (2008a) found that the 9R/9R genotype on DAT1 is negatively associated with the number of sex partners of adolescents compared to the 10R genotype. In addition, the 10R genotype on DAT1 is found to be generally related to more risky health behaviors (Guo et al., 2008b). The authors further find that particularly the 30bp VNTR region (Variable number of tandem repeats) on the Monoamine oxidase A gene (MAOA, x- chromosomal), the 40-bp VNTR region on DAT1 and the Taq1 polymorphism in DRD2 (dopamin receptor gene 2) are significantly associated with serious violent delinquencies, and interact with family and school circumstances as well as with friendship networks. In addition, Shanahan et al. (2008) found associations of DRD2 with educational continuation and social capital, again on basis of the “National Study of Adolescent Health.”

Due to the dramatically falling costs of sequencing and SNP genotyping, along with the development of new, fast and cheap sequencing methods such as the “nanopore sequencing” (https://www.nanoporetech.com), we expect a substantial expansion of candidate gene approaches in behavioral anthropology.

In addition to “candidate genes approaches,” owing to the availability of whole genome sequences we also expect “genome-wide association studies” to grow in importance.

### Genome wide association studies (GWAS)

GWAS are usually based on a large number of genome-wide sampled single nucleotide polymorphisms (SNPs), categorical or continuous phenotypically variables, and some control variables. The aim of a GWAS is to identify alleles (typically SNPs or combinations of SNPs) that are associated with a certain phenotypical trait. So far, GWAS have mostly been used in medical genetics to identify the genetic basis of diseases. The most severe limitation of GWAS is that they tend to explain only a small proportion of the phenotypical variance (even for traits known to have a strong genetic basis). This is because most phenotypical traits are multigenic or pleiotropic. Hence, the results of a GWA should not be over-interpreted, and lab and *in “silico”* studies should follow to clarify the found associations.

Recently, SNPs associated with the human facial form (Liu et al., 2012), depression (Converge Consortium, Cai et al., 2015), educational attainment (Rietveld et al., 2013), obesity (Hedman et al., 2014), height (Berndt et al., 2013), schizophrenia; [(Schizophrenia Psychiatric Genome-Wide Association Study (GWAS) Consortium, 2011)] and many more have been found.

Statistical testing in GWAS includes association tests for a “disease trait,” comparing allele frequencies between cases and controls, or various tests for quantitative traits; a detailed description of the statistical tests involved in GWAS studies is available in one of the most widely used software packages for GWAS studies: “PLINK” http://pngu.mgh.harvard.edu/purcell/plink/anal.shtml. PLINK is a commando line orientating program running in Linux, Mac and Windows, that reads genetic data in a “spreadsheet form” [e.g., pedigree information, sex, phenotype, and genetic loci (mostly SNPs) in rows, and individuals in lines]. Additionally, a map file provides information on the chromosome, the name of a locus (usually “reference cluster IDs”- rs numbers http://www.ncbi.nlm.nih.gov/SNP/get_html.cgi?whichHtml=how_to_submit#REFSNP), the position of a locus on a chromosome in base pairs, and available genetic distances in centimorgan. Plink can be used for data handling and conversion tasks, such as minimal allele frequency filtering, calculation of inbreeding coefficients, and GWA studies. Plink regresses genotypes and confounding variables on a categorical trait as well as on a quantitative trait, and returns the loci associated with a trait on basis of a “genome wide significance” (usually in human studies *p* < 10^−8^). In addition to Plink, programs such as for instance GENABLE (R- toolbox) also provide the tools for performing a GWAs.

The results of a GWA study are usually several SNPs that are significantly “statistically associated” with a trait. The high number of genetic loci and subjects involve the “risk” that, due to multiple testing, false positive signals may arise. Therefore, a certain *p*-value threshold is defined (in studies with human subjects usually *p* < 10^−8^). After identifying one or more loci associated with a certain trait, “detective work” often starts in the lab but also *in “silico”* using various online tools to identify the molecular and physiological function of the SNPs found being associated with a certain trait.

A very high number of participants is needed for a GWA study, and the sequencing effort is also high. GWAS are therefore currently restricted to major international consortia. Nonetheless, many data from GWAS are meanwhile available for free-use by the international scientific community. The genetic data samples by the “Health and Retirement Study (HRS- http://hrsonline.isr.umich.edu/index.php?p=gwas&jumpfrom=DD) and the personal genome project (http://www.personalgenomes.org/harvard), where individuals post their genotype data along with many phenotypical information are an impressive example of datasets suitable for GWAs studies. For the personal genome project the only restriction is the variety of genomic-formats, e.g., before working on the data, they have to be harmonized and transformed to a standard format such as PLINK.

GWAS, however, also have several limitations (a comprehensive review can be found in Visscher et al., 2014). GWAS mainly report correlations between genetic loci and certain phenotypes, but very often the biological relevance of such traits remains unknown (McClellan and King, 2010). Hence, the GWAS is unable to proof that a genomic variant actually causes a certain phenotype but can only give a hint of a potential gene/phenotypic association. In addition, due to the LD structure of the human genome, GWAS are designed to detect associations with causal variants that are relatively common in a population, and discovery rate (Visscher et al., 2014) increases with sample size (increasing the risk of false negative results). Furthermore, in many studies, the variance explained by GWAs is often relatively low (reviewed in Visscher et al., 2014).

Apart from these limitations, however, GWAS may become a powerful tool, in particular, if the biological functioning of loci detected by GWAS can be explained in later studies. In fact, GWAS have led to new discoveries about genes and pathways involved in several complex traits amongst others, depression (The CONVERGE consortium., 2015), schizophrenia [Schizophrenia Psychiatric Genome-Wide Association Study (GWAS) Consortium, 2011], and educational attainment (Rietveld et al., 2013).

### Epigenetics

Importantly, not only the nucleotide sequence itself but the pattern of gene expression creates the phenotype and thus, provides variation for selection. This calls for considering the epigenome, which regulates gene activity, as well. Moreover, the epigenome is subject to environmental influences, both internal and external. Integrating epigenetic information therefore, includes environmentally induced effects on the phenotype in the analyses. By mediating the interactions of the environment with the genome, epigenetic regulation enables a degree of sensitivity to fluctuating environmental conditions that greatly exceeds the much slower process of mutation and selection (LaFreniere and MacDonald, 2013).

The epigenome is a biochemical system that regulates gene expression without altering the nucleotide sequence. The main components of the epigenome are chromatin modifications that modulate DNA accessibility for the transcriptional apparatus such as DNA methylation and histone acetylation. DNA methylation, which is typically associated with down-regulation of gene expression, is the most accessible and best characterized epigenetic mark. The epigenetic control of gene activity, however, is much more complex, including besides several other histone modifications also non-coding RNAs.

There are several approaches to analyze the variation of epigenetic marks among individuals and populations, most of which focus on DNA methylation. In addition to candidate gene approaches, which address the epigenetic regulation of specific genomic regions, high-throughput techniques are now available to enable epigenome-wide association studies (Mill and Heijmans, 2013). Epigenetic modifications are tissue-specific, in contrast to genetic studies. This makes the choice of tissue a major challenge in epigenetic studies, posing basic questions about comparability and functional significance of epigenetic marks among different tissues.

As an example, Lam et al. (2012) used peripheral blood cells to measure DNA methylation in promoter regions of more than 14,000 genes. They characterized epigenetic variation and identified demographic and psychosocial factors co-varying with DNA methylation patterns.

### Other approaches

Only few fields are growing as rapidly as molecular biology, its many branches open the door for new studies in behavioral anthropology. Particularly two approaches seem of certain interest for behavioral anthropology:

- **Spousal Data**: Mating has always been a focus of behavioral anthropology. Numerous studies over the last 30 years investigated the criteria behind how individuals choose a mate, ranging from attractiveness, body composition, homogamy to personality dimensions, and many more. With the emergence of huge genomic databases which also cover individual relationships, the potential genomic basis and genomic consequences of human mating patterns can be studied. The questions related to how we choose individuals include genetic similarity (a “pilot study” has been published based on the HRS data last year; Domingue et al., 2014), whether there are loci on which couples tend to be more similar (for instance, genes involved in human behavior would be very interesting) or dissimilar (such as the “MHC”-complex), or whether spouses have a comparable mutation load or epigenetic pattern. This could be done technically with the help of two approaches: (i) applying GCTA (http://cnsgenomics.com/software/gcta/), a program that has been primarily build to estimate the variance explained by all SNPs of a genome for a complex trait (see below), or (ii) by KING (http://people.virginia.edu/~wc9c/KING/), which provides family relationship coefficients.
- **Twin Studies—Estimation of the proportion of the genetic predisposition of a trait**: For a long time, biologists and psychologist have been deeply interested in the heritability of traits, which has frequently been investigated using twin-studies (Boomsma et al., 2002; Plomin et al., 2008). On the basis of twins studies it has been found that for instance brain measures are heritable to a large extend (Jansen et al., 2015). This holds also true for aggression (Rhee and Waldman, 2002), “general” personality (Tellegen et al., 1988), economic risk taking (Zhong et al., 2009), economic game responder behavior (Wallace et al., 2007), pro- social behavior (Knafo and Plomin, 2006), and many more. A twin study combined with a candidate gene approach has further shown that monozygotic twins correlate more strongly in the timing of first sex than dizygotic twins and that individuals with a polymorphism in the Dopamine Receptor Gene 4 (DRD 4) have a higher chance of an earlier first intercourse (Guo and Tong, 2006). Twin studies, however, always have the problem to deal with the blurring of the results due to shared environmental influences. But statistical genetics now provides completely new approaches such as for instance the above-mentioned GCTA developed by Yang et al. (2010) to estimate the variance explained by all SNPs of a genome for a complex trait. A great advantage over classical twin studies, is that by regressing via some kind of “mixed model of genome wide SNPs of unrelated individuals on a trait, the variance explained by these SNPs can be estimated, making it possible to estimate to what proportion genetics contributes to that trait. It is safe to say that this approach will have a deep impact on behavioral genetics. With this approach it is also possible to estimate the genetic co-variance between two complex traits and thus, estimate the genetic overlap between those traits (Visscher et al., 2014). Tropf et al. (2015), for instance, used this method to estimate the genetic co-variance of number of children born and age at first birth. Such an approach may be also used to estimate the genetic response to selection.
- **Gene Editing**: Very recently, CRISPR system (clustered regularly interspaced short palindromic repeats; Jinek et al., 2012) has been discovered, enabling the direct manipulation of DNA sequences. CRISPR are segments of prokaryotic DNA containing short repetitions of base sequences, part of a prokaryotic immune system. Developed for “gene editing,” CRISPR is able to recognize, cut and insert genetic elements. For example, the genomes of zebra-fish and Drosophila have been successfully edited by the CRISPR system (Hwang et al., 2013; Port et al., 2014). We think it will be possible to test the behavioral consequences of genetic loci associated with a behavioral trait in an animal model, involving precisely editing the DNA sequence of that animal and observing the behavioral consequences of that genetic loci directly. This approach is simpler and cheaper than for instance using a knockout mice. As the CRISPR/Cas system is a very recently discovered tool, many more applications will follow soon.

## Conclusion

We hope to have convincingly demonstrated that—as in other fields of biology—molecular biology will also change behavioral anthropology, evolutionary psychology, evolutionary sociology, and bio-sociology fundamentally: Due to the growing number of discoveries of genes, genomic regions, SNPs regulative elements (identified by GWAs and candidate gene approaches) that are related to human behavior, it will be increasingly possible to investigate the genetic basis of our behavior and psychology. In turn, knowledge on the “genetics of human behavior” will enable the investigation of evolutionary questions on mutation, selection, and adaptation in the human genome. Furthermore, it will possible to “quantify” evolutionary questions using bioinformatics and statistical genetics.

## Author contributions

MF wrote the manuscript with a focus on genomics. SH wrote the manuscript with a focus on epigenetics.

### Conflict of interest statement

The authors declare that the research was conducted in the absence of any commercial or financial relationships that could be construed as a potential conflict of interest.
